# Investigation of Dynamic Viscoelastic Asymmetric Response of PA6 Film Based on Fractional Rheological Model

**DOI:** 10.3390/polym16172485

**Published:** 2024-08-30

**Authors:** Bowen Li, Guangkai Liao, Yuankang Li, Zhenyan Xie, Lingna Cui, Kaikai Cao, Yuejun Liu

**Affiliations:** 1 Key Laboratory of Advanced Packaging Materials and Technology of Hunan Province, Hunan University of Technology, Zhuzhou 412007, China; bowenli1125@163.com (B.L.); lyk05112020@163.com (Y.L.); xzy045@foxmail.com (Z.X.); lncui1102@126.com (L.C.); 2 Zhuzhou Times Engineering Plastics Industrial Co., Ltd., Zhuzhou 412008, China; caokk@csrzic.com

**Keywords:** PA6 film, dynamic viscoelasticity, time–temperature superposition, asymmetric dynamic response, fractional rheological model

## Abstract

Polyamide 6 (PA6) film as a typical viscoelastic material, satisfies the time–temperature superposition (TTS), and demonstrates obvious dynamic strain amplitude and frequency correlation under dynamic load. The investigation of the dynamic mechanical behavior of PA6 film is essential to ensure the safety of these materials in practical applications. In addition, dynamic mechanical property testing under conventional experimental conditions generally focuses on the short-term mechanical performance of materials. Therefore, the dynamic viscoelasticity of PA6 film was tested using a dynamic thermo-mechanical analyzer (DMA) in this study, and the complex modulus master curve was constructed based on time–temperature superposition (TTS) to realize the accelerated characterization of long-term mechanical properties. Furthermore, according to experimentally obtained asymmetric characteristics of the Cole–Cole diagram and the loss modulus master curve of the PA6 film, the parameter distribution of the fractional Zener model and the modified fractional Zener model were compared, and the asymmetric dynamic viscoelastic response of PA6 film under different conditions was systematically investigated using these models. The results indicate that the modified fractional Zener model can truly describe the dynamic asymmetric characteristics of PA6 film, verify the feasibility and advantages of the modified fractional rheological model, and provide some theoretical guidance for exploring the tensile rheological mechanism of PA6 film.

## 1. Introduction

Polyamide 6 (PA6) film is widely used in fields such as food packaging, biomedical applications, and industrial production due to its high mechanical strength and exceptional physical and chemical stability [1,2,3]. As a typical thermorheologically simple material following the time–temperature superposition principle, the viscoelasticity of PA6 film exhibits significant dynamic strain amplitude and frequency dependence under dynamic loading [4,5], which has an important impact on its safety during use. If an accurate stress–strain constitutive relationship can be established, it would provide compelling information for studying the mechanical response of PA6 film, which is crucial for understanding the viscoelasticity and predicting the performance of the material [6]. Many researchers have conducted extensive experimental investigations on PA6 film and similar materials, demonstrating that its viscoelasticity has a significant time (frequency) and temperature dependence [7,8,9]. Therefore, it is necessary to investigate the dynamic viscoelasticity of PA6 film from the experimental and theoretical perspectives. At present, in the field of mechanics and engineering analysis, the viscoelastic constitutive model is usually constructed based on Riemann–Liouville-type fractional calculus. It avoids solving for limits by combining differential and integral terms, which proves convenient for addressing nonlinear or asymmetric problems [10,11]. A series of classical rheological constitutive models have been developed in the form of series, parallel, or combination of basic mechanical units (Hooke Spring, Newton Dashpot), such as Maxwell model, linear body model, Burgers model, and so on. Zhu et al. [12] found that the traditional model represented by the generalized Maxwell model is considered to be one of the models that can reasonably describe the static viscoelasticity of PA6 films, but there are limitations in capturing the experimental data under dynamic loading. Additionally, the model exhibits high computational complexity and fails to accurately describe the true dynamic viscoelasticity of the material. In order to overcome the limitations of conventional models, new fractional rheological models by introducing fractional derivative constitutive units or by adding extra fractional elements have been proposed to study the viscoelasticity of materials [13], such as the fractional Kelvin model, fractional Burgers model, and fractional Zener model.

Compared with the traditional model, the fractional rheological model typically requires fewer parameters and can more accurately reflect the true viscoelasticity of the material in a wider temperature and frequency range [14,15]. Li et al. [16] found that the Burgers model cannot effectively characterize the dynamic viscoelasticity of an asphalt mixture (AB), and the existing fractional derivative Burgers (FDB I) model has limitations in application and construction concepts. Therefore, starting from the derivation of the constitutive equations of the FDB I model, the second-order fractional derivative Burgers (FDB II) model is constructed by introducing a fractional Abel dashpot, and found that the master curve-fitting results no longer exhibit oscillatory characteristics as in the conventional Burgers model. Cai et al. [17] proposed an improved nonlinear fractional derivative generalized Kelvin model by replacing Newton dashpots with fractional springpot element and used this model to fit the stress relaxation data. The results indicate that the fractional derivative generalized Kelvin model can more accurately describe the nonlinear stress relaxation characteristics of raw lean coal anisotropy, and the parameters in the model better reflect the anisotropy of the sample. Alcoutlabi and Martinez-Vega [18] studied the viscoelasticity of amorphous polymer, constructed the extended fractional solid (EFS) model by adding a fractional springpot on the basis of the fractional Zener model, and successfully characterized the asymmetry of the Cole–Cole diagram of polymethyl methacrylate (PMMA), proving the feasibility of the modified model. Yin et al. [19] further validated the applicability of the modified fractional Zener model in characterizing the asymmetric dynamics of material viscoelastic behavior. In addition, more modified viscoelastic constitutive models have been proposed to better understand and predict the complex viscoelastic behavior of different materials in frequency-dependent experiments [20,21].

In this paper, the asymmetric response of PA6 film samples under dynamic loading was investigated by using a NETZSCH 303 dynamic thermo-mechanical analyzer (DMA), NETZSCH, Selb, Germany, for temperature scanning tests at different frequencies and frequency scanning tests at different temperatures. Complex modulus (*E*′,*E*″) and loss factor (tan*δ*) master curves were constructed based on two TTS-related transformations, the Williams–Landel–Ferry (WLF) model [22] and the Arrhenius law [23], to realize the understanding and prediction of long-term mechanical properties of PA6 films. The dynamic asymmetric characteristics of the Cole–Cole diagram and loss modulus master curve of the material were obtained according to the experimental data. At the same time, the modified fractional Zener model was further derived by incorporating a fractional springpot with a fractional order of *β* into the original fractional Zener model. Finally, the parameter distributions of the above two models were compared and analyzed, and the experimental data were fitted to systematically investigate the applicability and advantages of the modified fractional Zener model in characterizing the dynamic viscoelastic asymmetric characteristics of PA6 film. This work provides some theoretical guidance for the application of the fractional viscoelastic constitutive model to explore the tensile rheological mechanism of PA6 film.

## 2. Experimental and Methods

### 2.1. Materials and Sample Preparation

Polyamide 6 raw material with a purity of 99.9% was purchased from Hunan Yuehua Chemical Co., Ltd. (Hengyang, China). The main physico-chemical characteristics of PA6 are listed in Table 1.

The raw material slices are initially placed in the electric constant temperature blast drying oven, placed at 80 °C for 8 h to remove surface moisture, then transferred in a vacuum drying oven at 120 °C for 12 h. The vacuum gauge should be monitored every two hours; if significant changes are observed, the vacuuming process should be repeated. Furthermore, the dried PA6 raw material is melt-extruded by a single-screw extrusion casting machine (FDHU35, Guangzhou General Experimental Analytical Instrument Co., Ltd., Guangzhou, China) to obtain a casting film with uniform thickness and no impurities. PA6 slices are introduced into the extruder through the feed cylinder and are driven by a single screw with a diameter of 3 cm and a pitch of 0.3 cm through the four temperature zones of the machine, which are the feed zone, the transition zone, the mold head zone, and the mold lip zone. Each temperature zone is subdivided into several smaller regions to ensure uniform heating and complete melting of the material. The parameters of the temperature zones are shown in Table 2.

**Figure 1:**
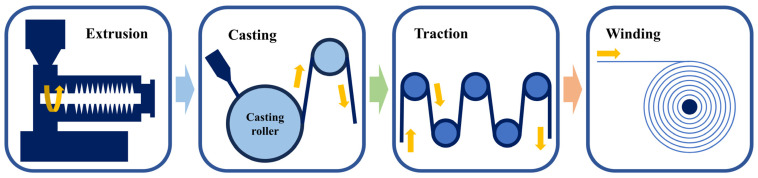
PA6 cast film preparation process.

The temperature in the feed zone is set close to the melting point of PA6 to prevent rapid melting of the polymer surface while the interior remains insufficiently melted, which could lead to channel blockage. In the subsequent temperature zone, the temperature is maintained approximately 30 °C above the melting point of PA6 to ensure uniform flow of the melt driven by the single screw. Additionally, the screw speed is set to 30 rpm to control the degree of stretching of the film in the casting direction, ensuring the film is as uniform as possible and reducing the degree of anisotropy. After extrusion, the PA6 melt is cooled on a casting roll with a set temperature of 30 °C, and then the casting film with a thickness of 0.250–0.300 mm is obtained by traction and winding. For convenience, the casting direction is referred to as the machine direction (MD), while the vertical casting direction is termed the transverse direction (TD). The process for preparing PA6 cast film is illustrated in Figure 1.

### 2.2. Dynamic Mechanical Analysis Test

Dynamic mechanical analysis refers to the technique of applying alternating loads on the material to measure the corresponding strain response [24]. The complex modulus can be expressed as E* = *E*′ + *iE*″, where the storage modulus (*E*′) and loss modulus (*E*″) reflect the rigidity and viscosity of the sample, respectively. The loss factor (tanδ) of the material is represented by *E*″/*E*′, that is, tanδ = *E*″/*E*′. 

Dynamic frequency scanning and multi-frequency dynamic temperature scanning experiments of PA6 film are carried out on a dynamic thermo-mechanical analyzer (DMA 242 E, NETZSCH, Selb, Germany). The PA6 cast film was cut into test samples of 13.50 × 5.00 × 0.250 (±0.010) mm^3^, and the test direction was determined to be the MD direction. In order to determine the isoconfigurational test conditions (material in the same aging state), the material was rejuvenated using an annealing process, that is, heating the material above the *T*_g_, maintaining for a period of time, and then cooling to room temperature (25 °C) to reduce the internal residual stress and eliminate the thermal history. Due to the inherent variability in DMA testing and slight differences between samples, data from each set of analyses may exhibit minor fluctuations or variations. Consequently, each set of experiments was repeated more than three times, and a representative set of data was selected for further analysis to ensure the usability and scientific validity of the results.

(1) Dynamic frequency scanning test

Dynamic frequency scanning tests were conducted on PA6 film samples using DMA in the frequency range of 0.1 Hz to 100 Hz, and the scanning tests were carried out from low frequency to high frequency. The temperature was controlled by liquid nitrogen, adopting the step-up heating method, with a temperature range of −10 °C to 110 °C and a step increment of 10 °C. Prior to each temperature test, the specimens underwent a 10-min isothermal treatment to ensure thermal equilibrium between the internal and external temperatures of the samples. The variation curves of the storage modulus (*E*′), loss modulus (*E*″), and loss factor (tanδ) of PA6 film under different conditions across the frequency sweep range were recorded. Based on the experimental measurement and recording procedure, the dynamic loading strain amplitude in DMA was set to 0.1% to ensure that the material response remained in the linear viscoelastic region. This is because a smaller strain amplitude can ensure better measurement accuracy and reduce the influence of nonlinear effects, thereby making the experimental results more predictable and repeatable. In addition, the pre-strain is 0.4% and the absolute target amplitude is 20 μm to ensure that the sample can maintain a stable state during dynamic loading. The maximum dynamic force (F_dyn_) is 2.182 N, which is automatically adjusted by the machine based on the sample size in order to ensure that this value is within a controllable range, thereby preventing overload and potential damage to the test sample.

(2) Multi-frequency dynamic temperature scanning test

PA6 film samples were heated from 0 °C to 160 °C at a heating rate of 3 °C/min, and the loading frequencies were 1, 2, 3, 5, 10, 15, 20, 30, and 50 Hz, respectively. The curves of dynamic modulus (*E*′, *E*″) and tanδ versus temperature were recorded at different frequencies. Similarly, in order to ensure the rigor and validity of the experiments results, the experimental conditions were kept consistent across different variables. The dynamic loading strain amplitude in DMA was set at 0.1%, with a pre-strain of 0.4%, the absolute target amplitude was 20 μm, and the F_dyn_ was 2.182 N.

### 2.3. Time–Temperature Superposition (TTS)

The time–temperature superposition principle is that the effect of temperature on the modulus of the material can be equivalent by changing the loading time of the material. Such materials are often referred to as thermorheologically simple materials [25], and the relaxation time of the molecular chain has the same temperature dependence. There are typically two common methods for determining the long-term mechanical properties of materials: long-term mechanical testing and accelerated characterization [26]. Long-term mechanical testing involves directly measuring the mechanical properties of materials under long-term loading and temperature conditions, thereby delivering an accurate assessment of their performance over time. Unfortunately, this method has a long testing cycle, and general experimental equipment and environment are insufficient to meet the requirements. Accelerated characterization involves conducting short-term mechanical tests on materials and constructing long-term mechanical performance master curves based on the time–temperature superposition approach, which facilitates understanding and predicting of the long-term mechanical properties of materials. However, this method still has limitations as it is only applicable to simple materials and without considering the internal damage of materials.

(1) Williams–Landel–Ferry (WLF) equation

The free volume theory quantitatively describes molecular free volume changes within polymers. On this basis, the WLF incorporates temperature effects, especially concerning the glass transition, thereby providing a more comprehensive explanation of the rheological behavior and dynamic properties of high-polymer materials under varying temperature conditions. According to the free volume theory, the viscosity (*η*) and free volume fraction (*f*) of a material should satisfy the Doolittle equation [27], Equation (1):

(1) $$\ln\eta =\ln A+B\left(\frac{1}{f}-1\right)$$

where *A*,*B* are material constants.

According to time–temperature superposition, the mechanical behavior of viscoelastic materials at different time scales will also change with the change of temperature. Furthermore, it is assumed that the free volume fraction of the material has a linear relationship with temperature, and is described as follows:

(2) $$f=f_{0}+\beta_{T}(T-T_{r})$$

where, *β*_T_ is the thermal expansion coefficient, *T*_r_ is the reference temperature, and *f*_0_ is the free volume fraction of the material under *T*_r_. The time–temperature superposition horizontal shift factor is denoted as *a*_T_ = *η*/*η*_0_, *η*_0_ is the viscosity of the material under *T*_r_, *η* is the viscosity of the material under temperature *T*, and the horizontal shift factor (log*a*_T_) is expressed as:

(3) $$\log a_{T}=\frac{B}{2.303f_{0}}\left(\frac{1}{f}-\frac{1}{f_{0}}\right)$$

According to Equation (2), Equation (3) can be further expressed as:

(4) $$\log a_{T}=-\frac{B}{2.303f_{0}}\left[\frac{T-T_{r}}{(f_{0}/\beta_{T})+T-T_{r}}\right]=-\frac{C_{1}(T-T_{r})}{C_{2}+(T-T_{r})}$$

where *C*_1_ = *B*/2.303*f* and *C*_2_ = *f*_0_/*β*_T_ are material parameters. Equation (4) is the famous WLF equation [22], which has been widely used in the temperature-related study of viscoelastic mechanical behavior of polymers. Specifically, the WLF equation considers the influence of temperature on polymer chain diffusion and mobility rates through parameters *C*_1_ and *C*_2_, describing the viscoelastic behavior of high polymer materials across different temperatures. Compared to the free volume theory alone, this approach more effectively addresses the predictive needs for material performance in practical engineering applications.

(2) Arrhenius law

Almost all temperature-dependent laws of molecular motion obey the Arrhenius law, and the Arrhenius equation is expressed as follows:

(5) $$\tau =A\exp\left(\frac{E_{a}}{RT}\right)$$

where *τ* is the physical quantity of the system, *A* is a constant, *E_a_* is the activation energy, *R* is the molar volume constant, and *T* is the thermodynamic temperature. The time–temperature superposition horizontal shift factor is denoted as *a*_T_ = *τ*/*τ*_0_. Where *τ*_0_ is the relaxation time of the polymer under *T*_r_ and *τ* is the relaxation time under temperature *T*, then the horizontal shift factor is expressed as:

(6) $$\log a_{T}=\frac{1}{2.303}\frac{E_{a}}{R}\left(\frac{1}{T}-\frac{1}{T_{r}}\right)$$

Comparing Equation (4) and Equation (6), let the parameters *C*_2_ = *T*_r_, *C*_1_ = (1/2.303)× *Ea*/*RT*_r_), it can be found that the Arrhenius and WLF are equivalent.

### 2.4. Thermo-Rheological Properties of PA6 Film

**Figure 2:**
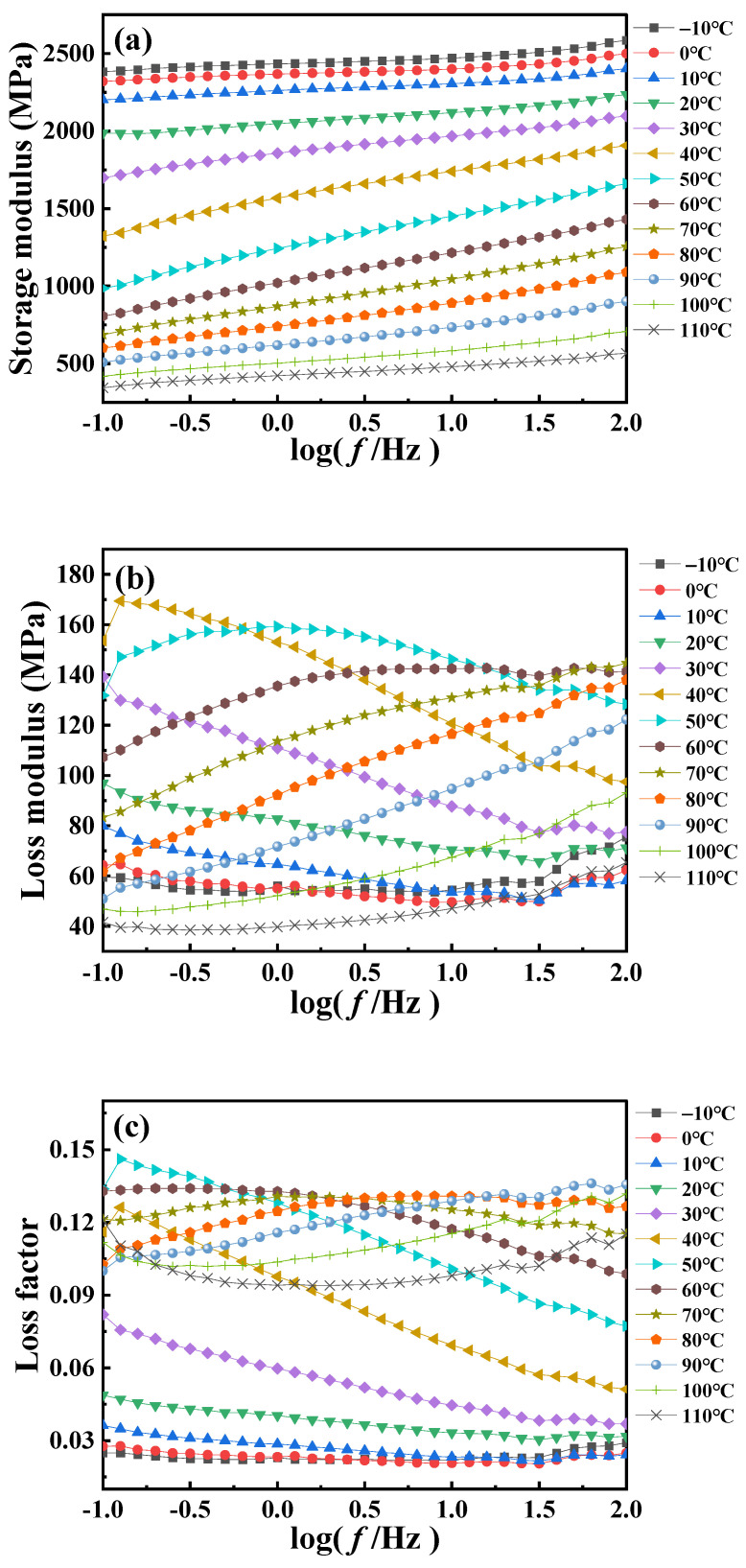
The frequency-scanning DMA curves of PA6 films at different temperatures: (**a**) storage modulus, (**b**) loss modulus, (**c**) loss factor.

Figure 2 is the dynamic frequency scanning DMA curve of PA6 film at different temperatures. The results show that the dynamic modulus and loss factor of the PA6 film exhibit pronounced dependence on loading frequency and temperature. It is observed from Figure 2a that the PA6 film remains stable as a whole when the frequency changes, indicating that the response of the material is in the linear viscoelastic range under this experimental condition. However, the amplitude of the *E*′ with frequency is relatively obvious at the temperature near the glass transition, while it exhibits a reduced sensitivity to frequency at low temperature or high temperature. This can be explained by the fact that the glass transition process involves large-scale synergistic molecular motions, leading to significant changes in the internal structure of the polymer material and thus significantly affecting the dynamic mechanical properties of the material. In contrast, at low or high temperatures, the motion of the polymer chains is relatively stable, resulting in an insignificant response to frequency. The above conclusion is further supported by Figure 2b, from which it can be observed that the *E*″ is lower at both low and high temperatures and remains relatively constant with increasing frequency. This is because at low temperatures the relatively restricted motion of PA6 molecules results in a lower loss modulus, whereas at high temperatures, although there is an increase in molecular motion, it may also result in a relatively low loss modulus due to the higher energy state. At high frequencies, the interaction between PA6 molecules exhibits not only elastic behavior (energy storage modulus) but also viscous behavior (loss modulus). Near the *T*_g_, this viscoelastic behavior becomes more pronounced due to the rapid molecular motion, resulting in increased energy dissipation and internal friction.

**Figure 3:**
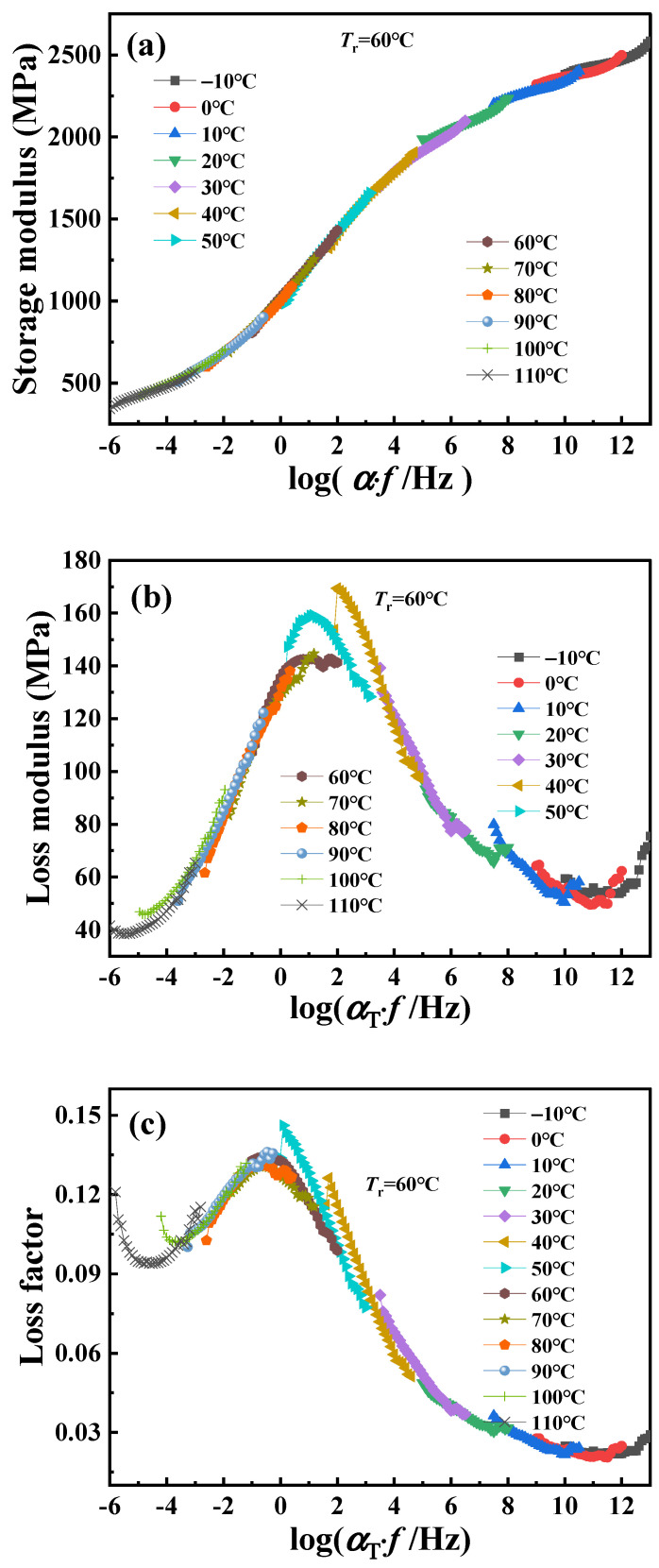
The master curve of dynamic mechanical properties of PA6 film at reference temperature of 60 °C: (**a**) storage modulus, (**b**) loss modulus, (**c**) loss factor.

**Figure 4:**
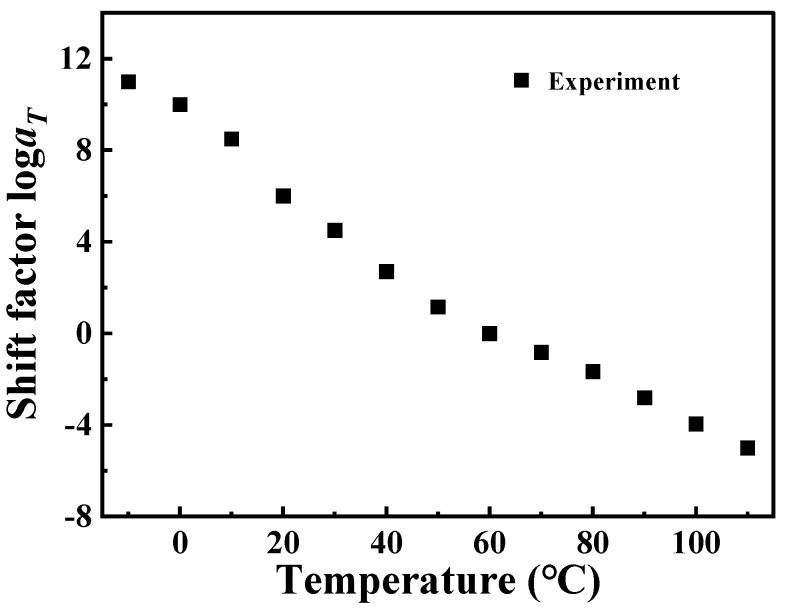
The relationship curve between horizontal shift factor and temperature.

Using time–temperature superposition, the glass transition temperature of PA6 is selected as the reference temperature (*T*_r_ = 60 °C). Each curve at different temperatures in Figure 2 is shifted horizontally until all curves merge into a smooth master curve, as shown in Figure 3. These master curve results reflect the dynamic mechanical behavior of PA6 film in a wide range of frequencies covering about 19 decades. At the same time, it also proved that PA6 film is a thermorheologically simple material. The relationship between horizontal shift factor and temperature is shown in Figure 4.

**Figure 5:**
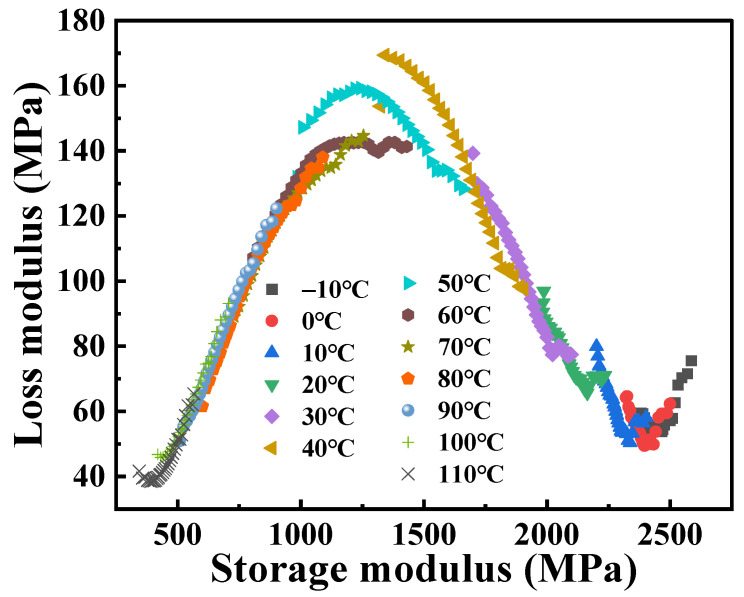
Cole–Cole diagram of PA6 film at different temperatures.

The real part (*E*′) and the imaginary part (*E*″) of the complex modulus derived from the dynamic frequency scanning results at different temperatures are taken as the *X*-axis and the *Y*-axis, respectively, and the Cole–Cole diagram of the PA6 film is obtained [28], as shown in Figure 5. It can be observed that the Cole–Cole diagram exhibits significant asymmetry, but the curves overlap poorly at low temperatures. This can be explained by the fact that the PA6 molecular structure is rigid at lower temperatures; the dynamic response is inflexible. Additionally, it should be noted that the slope of the Cole–Cole diagram remains essentially constant in the high-temperature zone. This is consistent with the fact that physical aging cannot be observed in this temperature region because polymers are in thermodynamic equilibrium at temperatures well above the *T*_g_. As the temperature approaches the *T*_g_, the modes of motion and energy dissipation within the material change, resulting in an increase in the loss modulus, which manifests itself as a smoother curve. 

The horizontal shift factor can also be determined by the Arrhenius equation. Firstly, *T*_g_ at different loading frequencies was determined. In the DMA curve, the temperature at which the maximum value of tan*δ* occurs corresponds to the *T*_g_ of the polymer, indicating that the energy loss of the material reaches its maximum at this temperature, which is directly related to a significant increase in the mobility of the molecular chains [29]. The equation for calculating the activation energy of materials according to the Arrhenius law is as follows:

(7) $$f=f_{0}\exp\left(\frac{-E_{a}}{RT_{g}}\right)$$

where *f*_0_ is a constant. Taking the logarithm of Equation (7) yields:

(8) $$\ln f=\ln f_{0}-\frac{E_{a}}{R}\frac{1}{T_{g}}$$

**Figure 6:**
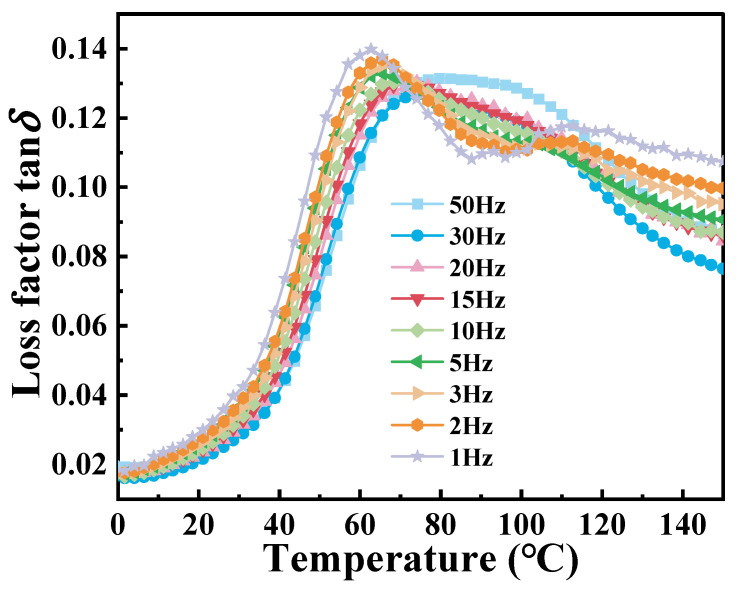
Loss factor of PA6 film multi-frequency temperature scanning DMA curve.

The multi-frequency dynamic temperature-scanning DMA curves of PA6 film at different frequencies are shown in Figure 6. It can be observed that the peak of tan*δ* gradually shifts to the right with the increase in frequency. This shift can be explained as that the movement of the molecular chain of the material is limited at high frequencies, resulting in reduced intermolecular friction, which is accompanied by low energy dissipation. During the transition from low to high frequencies, the tan*δ* curve gradually evolves from two distinct peaks to a broader single peak. The reason is that the micro-domain crystallization behavior occurs due to the influence of the thermal history of the material at low frequency. At higher frequencies, molecular chains do not have sufficient time to respond to strain, restricting molecular mobility and causing different modes of motion to merge into a broader peak. Table 3 presents the corresponding *T*_g_ values at different frequencies.

**Figure 7:**
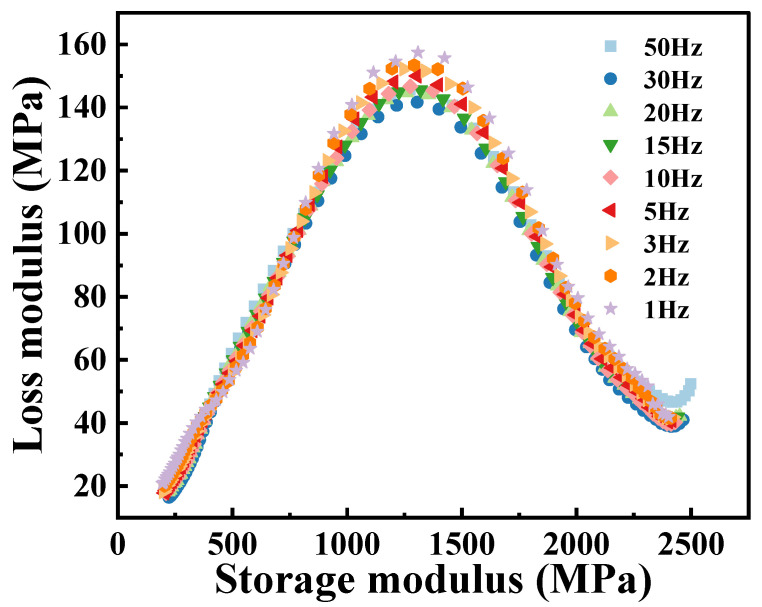
Cole–Cole diagram of PA6 film at different frequencies.

The Cole–Cole diagram of the PA6 film obtained from temperature scanning at different frequencies, as shown in Figure 7, similar to the shape of the curve exhibited in Figure 5, and also exhibits classical asymmetric characteristics. In addition, the loss modulus at different frequencies varies relatively around the peak of the Cole–Cole diagram (corresponding to the temperature region of *T* = 60 °C in Figure 5), indicating that the dynamic mechanical response of PA6 films is more sensitive to frequencies near the glass transition temperature. At low frequency conditions (1 Hz), the molecules of the material have more time to perform complex motions in respond to external forces, thereby more effectively converting energy into heat, resulting in a higher loss modulus. On the contrary, at high frequencies (50 Hz), the degree of freedom of the molecular chain is limited, resulting in a smaller loss modulus. In the Cole–Cole diagram, the slopes at both ends of the curve are basically the same, indicating that the dynamic response of the PA6 film in both the low and high temperature regions has little dependence on the frequency. At lower temperatures, PA6 is in the glassy state, with its molecular chains in a “frozen” state, resulting in minimal velocity and amplitude of chain motion. However, due to the existence of *β* relaxation, the loss modulus of the material will increase, and the increase effect will be more obvious, especially at high frequencies. During the glass transition process, the dynamic response of the material is more affected by the molecular thermal motion, and the influence of *β* relaxation will gradually disappear or weaken. At this time, the movement mode and structural state of the PA6 internal molecular chain are quite different, resulting in obvious dynamic asymmetric characteristics. As the temperature further increases, the PA6 film transforms into the rubber state and its internal structure and molecular chain motion are almost the same, which leads to the consistent response at different frequencies, which is manifested as the coincidence of the Cole–Cole diagram in the high-temperature region.

**Figure 8:**
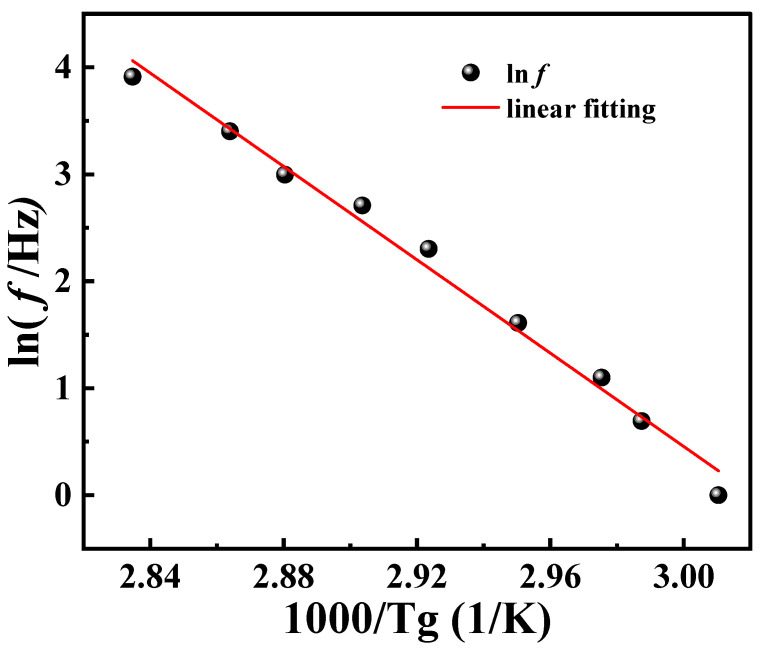
Arrhenius plot fitted by Equation (8).

**Figure 9:**
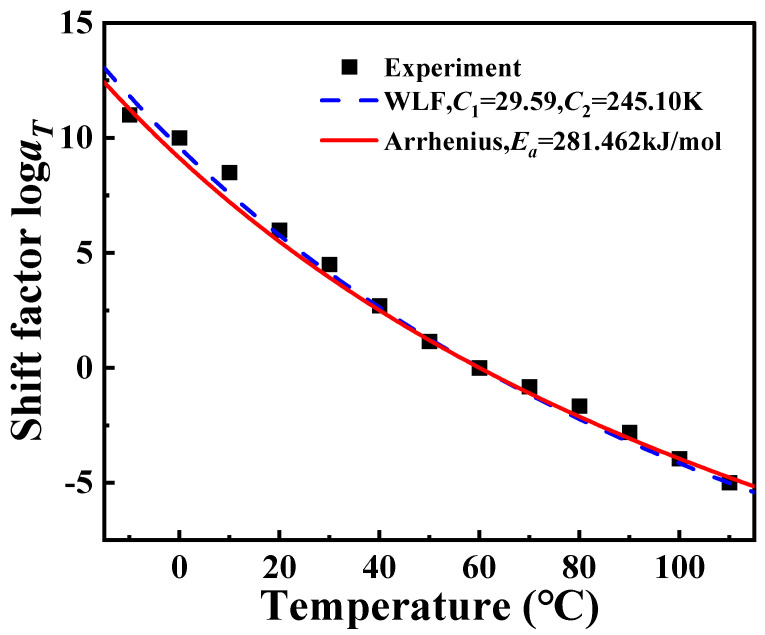
Horizontal shift factor model and experimental comparison.

According to Equation (8), the logarithmic frequency is linearly related to the reciprocal of *T*_g_. A linear fit was performed on the data presented in Table 2, as shown in Figure 8. Further, the *E_a_* of PA6 film is calculated to be 281.462 kJ/mol. Taking *E_a_* into Equation (6), log*a_T_* can also be calculated. Due to the single-use and non-recyclable nature of DMA test samples combined with the inherent microscopic variations among PA6 film samples, *T*_g_ measured at different frequencies may exhibit variability across tests. Consequently, this variability leads to alterations in the fitted curves, resulting in some degree of error in the calculated values of *E_a_*. However, these errors remain within an acceptable range and do not significantly impact the overall results. At the same time, the data in Figure 4 are fitted by Equation (4), and the parameters *C*_1_ = 29.59 K, *C*_2_ = 245.10 K are obtained, as shown in Figure 9. It can be observed that the log*a_T_* obtained by using Arrhenius, the results of WLF equation fitting, and the results of the horizontal shift factor of the experimental data are all in good agreement among the three, which leads to the conclusion that both Arrhenius and WLF can be used as research methods for the investigation of the rheological behavior of PA6 film.

## 3. Fractional Rheological Constitutive Model

### 3.1. Classical Fractional Zener Model

In the field of rheology, the Zener model is often used to study viscoelastic solids. By replacing the Newton dashpot in the Zener model with a fractional springpot [30,31], the fractional Zener model can be obtained. The fractional springpot mechanical response can be expressed by Equation (9) as follows:

(9) $$\sigma \left(t\right)=E^{1-\alpha }\eta^{\alpha }D^{\alpha }\varepsilon \left(t\right)=E\tau^{\alpha }D^{\alpha }\varepsilon \left(t\right)$$

where *E* is the elastic modulus, *τ* = *η*/*E* is the characteristic relaxation time of the material, *D*^α^ is the Riemann–Liouville fractional derivative, *α* is a fractional order, 0 ≤ *α* ≤ 1. It is worth noting that when α = 0 or α = 1, the fractional springpot is degraded to a Hooke spring element or a Newton dashpot element, respectively. The definition of *D*^α^ is:

(10) $$D^{\alpha }\left(f\right)=\frac{1}{\Gamma (1-\alpha )}\frac{d}{dt}\int_{0}^{t}\frac{f\left(s\right)}{t-s}ds,f\left(t=0\right)=0$$

where *Γ*(*x*) is the gamma function [32]:

(11) $$\Gamma \left(x\right)=\int_{0}^{\infty }\left(e^{-u}u^{x-1}\right)du,x>0$$

**Figure 10:**
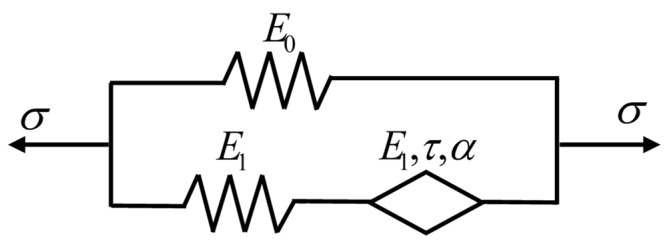
The fractional Zener model.

The fractional Zener model is composed of a fractional Maxwell body and a Hooke spring in parallel, as shown in Figure 10. The strain and stress of the Hooke spring in parallel with the fractional Maxwell body are *ε*_1_ and *σ*_1_, respectively, where *σ*_1_(t) = *E*_0_*ε*_1_(t). The strain of the spring and the fractional springpot in the fractional Maxwell body are *ε*_21_ and *ε*_22_, respectively, under the action of stress *σ*_2_, where *ε*_21_ = *σ*_2_/*E*_1_, *ε*_22_ = *σ*_2_/(*E*_1_*τ^α^D^α^*). Then, the total strain of the fractional Maxwell body is *ε*_2_ = *ε*_21_ + *ε*_22_, and the constitutive equation is obtained as follows:

(12) $$\sigma_{2}\left(t\right)=\frac{E_{1}\tau^{\alpha }D^{\alpha }}{1+\tau^{\alpha }D^{\alpha }}\varepsilon_{2}\left(t\right)$$

The total strain of the fractional Zener model is *σ* = *σ*_1_ + *σ*_2_, and the constitutive equation as follows:

(13) $$\sigma \left(t\right)=\left(E_{0}+\frac{E_{1}\tau^{\alpha }D^{\alpha }}{1+\tau^{\alpha }D^{\alpha }}\right)\varepsilon \left(t\right),\left(0\leq \alpha \leq 1\right)$$

where *E*_0_ is the elastic modulus of the equilibrium elastic Hooke spring, and *E*_1_ is the elastic modulus of the Hooke spring and the fractional springpot element in the non-equilibrium fractional Maxwell body.

Under the unit step load, by applying the Fourier transform to Equation (13), the real part (*E*′) and imaginary part (*E*″) of the dynamic modulus (Equations (14) and (15)) of the fractional Zener model are obtained:

(14) $$E^{\prime }\left(\omega \right)=E_{0}+\frac{E_{1}\tau^{2\alpha }\omega^{2\alpha }+E_{1}\tau^{\alpha }\omega^{\alpha }\cos\left(\alpha \pi /2\right)}{1+\tau^{2\alpha }\omega^{2\alpha }+2\tau^{\alpha }\omega^{\alpha }\cos\left(\alpha \pi /2\right)}$$

(15) $$E^{\prime \prime }\left(\omega \right)=\frac{E_{1}\tau^{\alpha }\omega^{\alpha }\sin\left(\alpha \pi /2\right)}{1+\tau^{2\alpha }\omega^{2\alpha }+2\tau^{\alpha }\omega^{\alpha }\cos\left(\alpha \pi /2\right)}$$

where the angular frequency *ω* = 2π*f*, the frequency is *f*.

**Figure 11:**
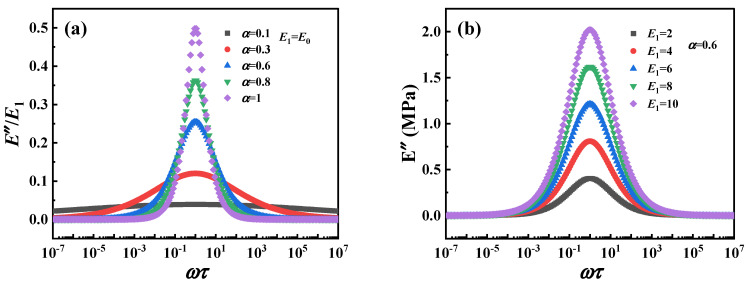
The normalized loss modulus of the fractional Zener model: (**a**) the influence of parameter α, (**b**) the influence of parameter *E*_1_.

**Figure 12:**
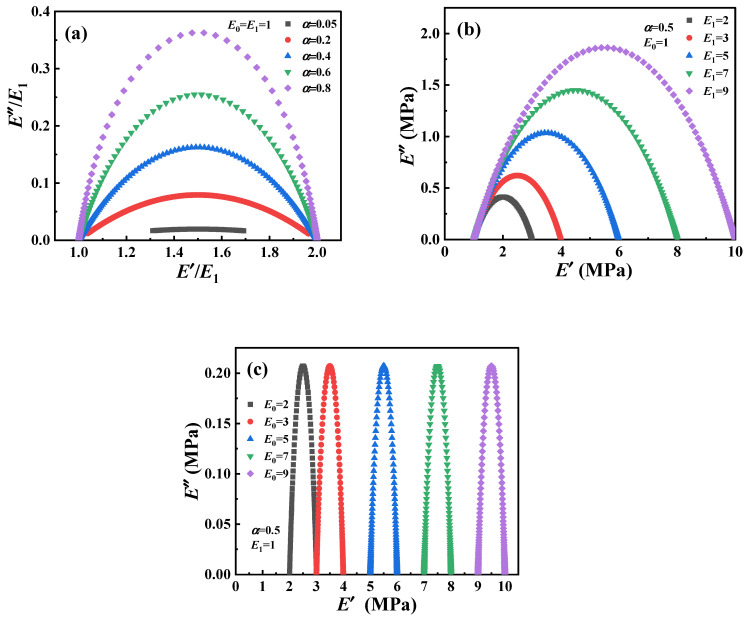
Cole–Cole diagram of fractional Zener model: (**a**) the influence of parameter α, (**b**) the influence of parameter *E*_1_, (**c**) the influence of parameter *E*_0_.

The normalized loss modulus (*E*″/*E*_1_) of the fractional Zener model and the parameter distribution of the Cole–Cole diagram are shown in Figure 11, respectively. It can be observed that the value of the *E*″/*E*_1_ decreases with decreasing *α*, indicating that α can describe the dynamic mechanical behavior of the material with low energy dissipation. The change of *E*_1_ reflects the response of the material in a wide frequency range, which also indicates that the fractional Zener model can understand and predict the viscoelastic response in a wide time range. On the other hand, the corresponding Cole–Cole diagram parameter distributions are shown in Figure 12 and can be clearly observed to be symmetrical features about the peaks. However, regardless of which parameter (*E*, *α*, *τ*) is varied, the fractional Zener model distribution curves of the loss modulus and Cole–Cole diagram show a completely symmetric character about the peaks, which is inconsistent with the previous experimental phenomenon.

In the fractional rheological constitutive model, the fractional order is a physical quantity describing the viscoelastic strength of the material. As parameter *α* tends to 0 or 1, and the macroscopic properties of the material tend to be ideal solid or ideal liquid, respectively. However, the fractional Zener model has only one fractional operator, which leads to the symmetry of the normalized loss modulus curve and the Cole–Cole diagram, and thus cannot reflect the real viscoelastic behavior of the material. In contrast, the actual dynamic viscoelastic response of PA6 film is asymmetric because the viscoelastic behavior response is different in long and short timescales. These findings emphasize the need to comprehensively consider the actual viscoelastic behavior of materials and its changes in time scales when developing more accurate fractional rheological models.

### 3.2. The Modified Fractional Zener Model

**Figure 13:**
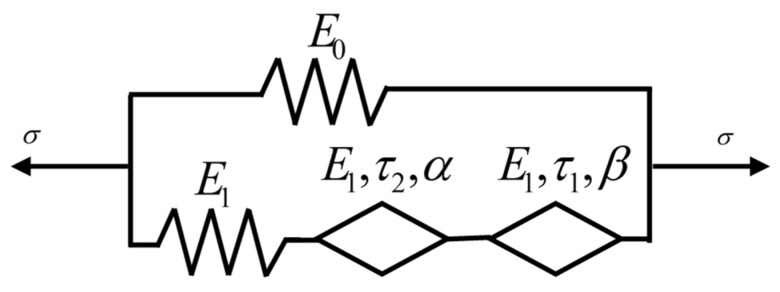
The modified fractional Zener model.

In order to be able to accurately describe the asymmetric response exhibited by the loss modulus master curves and Cole–Cole diagram of PA6 film, a fractional springpot of fractional order β is introduced into the fractional Zener model (Figure 10) to modify the original model, and the modified fractional Zener model is obtained, as shown in Figure 13. This model has three mechanisms: polymer elastic behavior, viscoelastic behavior at low temperature or high frequency, and viscoelastic behavior at high temperature or low frequency, and its constitutive equation is as follows:

(16) $$\sigma \left(t\right)=\left(E_{0}+\frac{1}{\frac{1}{E_{1}}+\frac{1}{E_{1}\tau_{1}^{\beta }D_{t}^{\beta }}+\frac{1}{E_{1}\tau_{2}^{\alpha }D_{t}^{\alpha }}}\right)\varepsilon \left(t\right),0\leq \alpha \leq \beta \leq 1$$

Under unit step load, the dynamic modulus of the model, (*E*×*iω*) = *E*′(*ω*) + *iE*″(*ω*), can be calculated by applying a Fourier transform to Equation (16), and obtained:

(17) $$E^{\prime }=E_{0}+\frac{E_{1}AB\left[AB+B\cos(\frac{\beta \pi }{2})+B\cos(\frac{\alpha \pi }{2})\right]}{A^{2}B^{2}+2AB\cos(\frac{\beta \pi }{2})\cos(\frac{\alpha \pi }{2})+2AB^{2}\cos(\frac{\beta \pi }{2})+2A^{2}B\cos(\frac{\alpha \pi }{2})+2AB\sin(\frac{\beta \pi }{2})\sin(\frac{\alpha \pi }{2})+A^{2}+B^{2}}$$

(18) $$E^{\prime \prime }=\frac{E_{1}AB\left[B\sin(\frac{\beta \pi }{2})+B\sin(\frac{\alpha \pi }{2})\right]}{A^{2}B^{2}+2AB\cos(\frac{\beta \pi }{2})\cos(\frac{\alpha \pi }{2})+2AB^{2}\cos(\frac{\beta \pi }{2})+2A^{2}B\cos(\frac{\alpha \pi }{2})+2AB\sin(\frac{\beta \pi }{2})\sin(\frac{\alpha \pi }{2})+A^{2}+B^{2}}$$

where A = ω^β^$\tau_{1}^{\beta }$, B = ω^α^$\tau_{2}^{\alpha }$.

**Figure 14:**
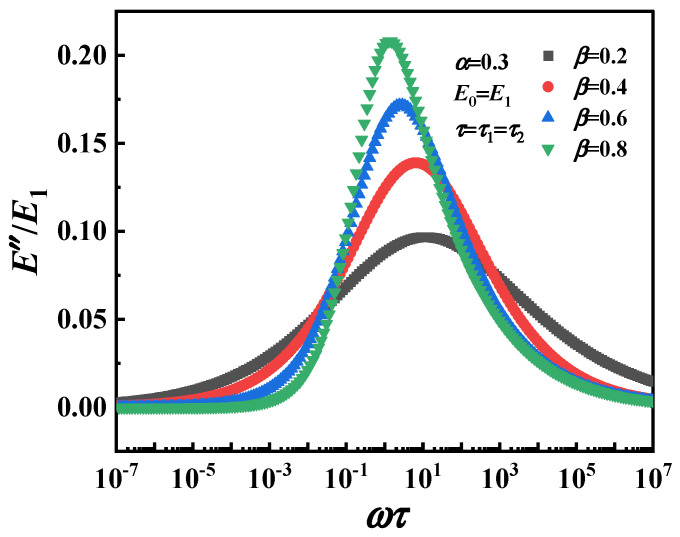
Normalized loss modulus *E*″/*E*_1_, for the modified fractional Zener model.

Figure 14 shows the parameter distribution of the fractional Zener model, the prediction of the normalized loss modulus as a function of frequency for different fractional order *β* values (0.2, 0.4, 0.6, 0.8) and fixed values other parameters (*E*, *α*, *τ*). It can be observed that the size and position of the peak are affected by parameter *β*, which is consistent with the evolution of the real modulus with frequency. At low frequencies, *E*″/*E*_1_ decreases with increasing *β*, which indicates that the springpot with fractional order *β* reflects the viscoelastic behavior at low frequencies or high temperatures. At high frequencies, the curves with *β* > 0.5 are highly coincident, which means that α (which, in this case, is constant) is related to viscoelastic behavior at high frequencies or low temperatures. Between these two regions, *E*″/*E*_1_ increases sharply with the increase in *β*, which is related to the glass transition anelastic manifestation.

**Figure 15:**
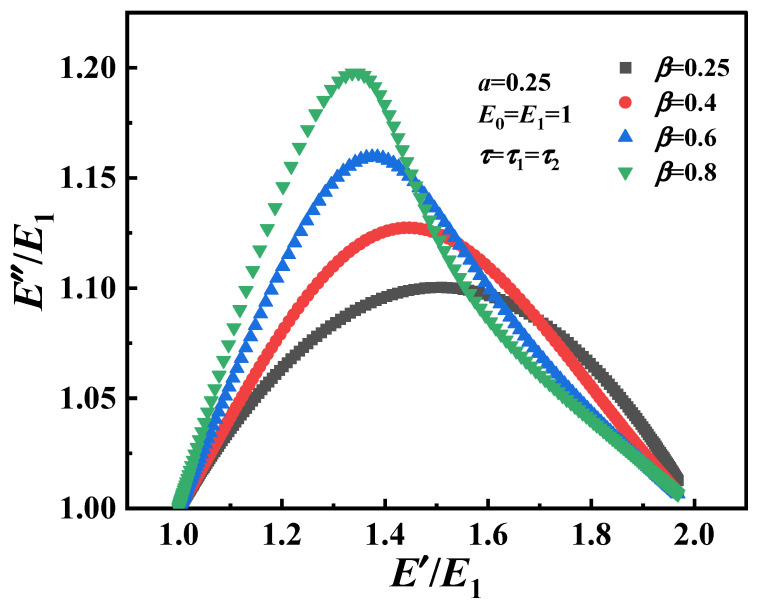
Cole–Cole diagram for the modified fractional Zener model.

The Cole–Cole diagrams (Equations (17) and (18)) of the modified fractional Zener model are shown in Figure 15, and the obvious asymmetric characteristics can be observed. On the other hand, the same behavior of parameters α and *β* at high frequency/temperature or low frequency/temperature are also obtained. Therefore, it can be considered that the modified fractional Zener model can predict the real viscoelastic response of fully (amorphous polymers) or partially (semi-crystalline ones) non-crystalline polymers, such as PA6, and this model can be further analyzed compared with the theoretical and experimental data, which was established in a large frequency range by dynamic mechanical testing methods.

### 3.3. Modeling of Dynamic Asymmetric Response of PA6 Film

**Figure 16:**
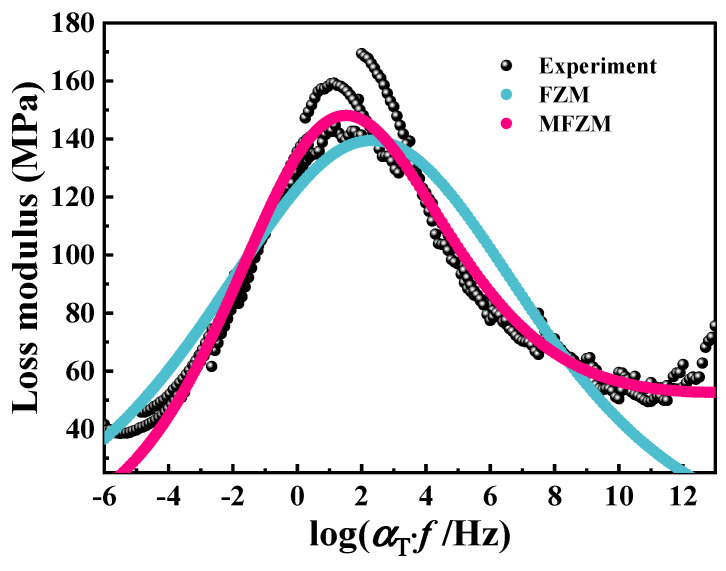
Master curve (60 °C) of loss modulus: model and experimental comparison.

**Figure 17:**
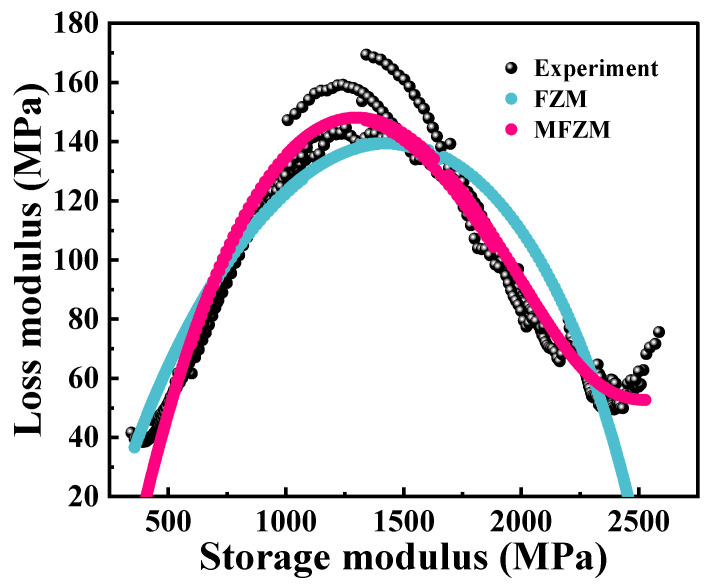
Cole–Cole diagram: model and experimental comparison.

The fractional Zener model and the modified fractional Zener model are employed to fit the normalized loss modulus master curve and Cole–Cole diagram of PA6 film constructed based on the time–temperature superposition principle, as shown in Figure 16 and Figure 17, to verify the applicability of the above two models. Obviously, the fractional Zener model exhibits a fully peak symmetry feature on the normalized loss modulus curve and Cole–Cole diagram, which cannot truly describe the dynamic viscoelastic behavior of PA6 film. The modified fractional Zener model can be used to model the dynamic viscoelastic asymmetric response of PA6 film. The parameters of the fractional Zener model are shown in Table 4 and Table 5 and the parameters of the modified fractional Zener model are shown in Table 6 and Table 7. The parameters α and *β* in the modified fractional Zener model depend on the thermal history of the polymer, and their values are less than 0.5, indicating that the properties of the PA6 film are closer to the macroscopic solid properties.

The above results show the consistency of the fractional-order theoretical method in a wide frequency and temperature range (from *T*_g_ -80 °C to *T*_g_ +50 °C). It is shown that the fractional rheological constitutive model method allows one to calculate the analytical theoretical equations of the real and imaginary parts of the dynamic modulus and that the relaxation time spectrum (H(*τ*)) can be obtained analytically. In addition, the parameters of the model are phenomenological parameters, which are not well connected to the actual physical properties of the material. Future research should focus on their molecular origin and rheological mechanism in the process of material deformation.

The fractional calculus approach gives predictions of the entire viscoelastic behavior of thePA6 film, and it can obtain the analytical equations for all viscoelastic functions. The model parabolic data describes the dynamic mechanical behavior of PA6 films in the linear viscoelastic range, and due to the relaxation and structural recovery of this type of polymer, there is a limit in the glass transition process.

## 4. Conclusions

In this study, the dynamic viscoelastic response of PA6 film was experimentally characterized over a wide frequency range and the asymmetric characterization of the dynamic modulus master curves (covering about 19 decades of frequencies) and Cole–Cole diagram were obtained. Further, the modified fractional Zener model is proposed by adding a springpot to the fractional Zener model, and the expressions for the constitutive relations for both models are derived. Finally, the modeling ability of the modified model was checked by the experimental results. The following conclusions were obtained:

(1) The dynamic viscoelasticity of PA6 films was tested using a dynamic thermomechanical analyzer (DMA) with multi-frequency temperature scanning and frequency scanning experiments at different temperatures, and the dynamic viscoelasticity showed significant dynamic strain amplitude and frequency correlation.
(2) The rheological properties of PA6 films were investigated using the WLF and Arrhenius law to verify that PA6 film is a thermorheologically simple material that satisfies the time–temperature superposition principle. Based on the time–temperature superposition principle, the dynamic modulus master curve and Cole-Cole diagram of PA film at the reference temperature of 60 °C were obtained, and the long-term mechanical behavior of PA6 film was accelerated by short-term experimental data. In addition, it was also demonstrated that the dynamic viscoelastic response of PA6 films is asymmetric.
(3) A comprehensive comparative analysis was conducted on the fractional Zener model and the modified fractional Zener model concerning their fitting capabilities for the asymmetric dynamic viscoelastic response of PA6 film. It was found that the fractional Zener model fails to accurately reflect the true viscoelastic behavior of PA6 film, whereas the modified fractional Zener model effectively captures the asymmetric dynamic viscoelastic response of PA6 film. At the same time, the advantages and feasibility of the original classical model correction based on fractional order theory were verified.

## Figures and Tables

**Table 1:** Physico-chemical characteristics of PA6.

Title	Symbols	Values	Units
Glass transition temperature	*T*_g_	60	°C
Density	*ρ*	1.158	g/cm^3^
Melt index	MI	3.8	g/min
Melting temperature	*T*_m_	220	°C

**Table 2:** The parameters of the temperature zones of the extruder casting machine.

Sample	Feed Zone (°C)				Transition Zone (°C)		Mold Head Zone (°C)			Mold Lip (°C)
	Zone 1	Zone 2	Zone 3	Zone 4	Zone 1	Zone 2	Zone 1	Zone 2	Zone 3	Zone 1
PA6	215	250	250	250	255	255	255	255	255	245

**Table 3:** *T*_g_ of PA6 film at different frequencies.

*f*/Hz	1	2	3	5	10	15	20	30	50
*T*_g_/K	332.18	334.74	336.09	338.94	342.05	344.40	347.17	349.17	352.77

**Table 4:** The parameters of the master curve (60 °C) of the loss modulus fitted by the fractional Zener model.

*E*_1_ (MPa)	*τ* (s)	*α*	*R* ^2^
2640.20	7.67 × 10^−4^	0.134	0.839

**Table 5:** The parameters of the Cole–Cole diagram fitted by the fractional Zener model.

*E*_0_ (MPa)	*E*_1_ (MPa)	*τ* (s)	*α*	*R* ^2^
219.21	2285.16	1.98 × 10^−3^	0.149	0.950

**Table 6:** The parameters of the master curve (60 °C) of the loss modulus fitted by the modified fractional Zener model.

*E*_1_ (MPa)	*τ*_1_ (s)	*β*	*τ*_2_ (s)	*α*	*R* ^2^
7641.90	1.64 × 10^−5^	0.206	3.69 × 10^−32^	0.186	0.976

**Table 7:** The parameters of the Cole–Cole diagram fitted by the modified fractional Zener model.

*E*_0_ (MPa)	*E*_1_ (MPa)	*τ*_1_ (s)	*β*	*τ*_2_ (s)	*α*	*R* ^2^
350.78	3738.52	4.50 × 10^−4^	0.209	5.45 × 10^−8^	0.023	0.976

## Data Availability

Data are contained within the article.

## Nomenclature

**Symbol**	**Definition or explanation**	**Symbol**	**Definition or explanation**
*R* ^2^	Model fitting degree	*C*_1_	WLF material parameters *C*_1_ = *B*/2.303*f*
*T*_g_	Glass transition temperature	*C*_2_	WLF material parameters *C*_2_ = *f*_0_/*β*_T_
*ρ*	Density	*τ*	Physical quantity of the system
MI	Melt index	*Ea*	Activation energy
T_m_	Melting temperature	*R*	Molar volume constant
MD	Machine direction (casting direction)	*τ*_0_	Relaxation time of the polymer under *T*_r_
TD	Transverse direction	log *f*	Frequency/Hz
*E**	Complex modulus *E** = *E*′ + *iE*″	σ	Stress
*E*′	Storage modulus	*ε*	Strain
*E*″	Loss modulus	*E*	Elastic modulus
tan *δ*	Loss factor	*α*	Fractional order, 0 ≤ *α* ≤ 1
Fdyn	The maximum dynamic force in DMA	*τ*	Relaxation time of the material, *τ = η/E*
*η*	Viscosity	*D* ^α^	Riemann-Liouville fractional derivative
*f*	Free volume fraction	*Γ*(*x*)	Gamma function
A	Material constants	*ω*	Angular frequency, *ω = 2πf*
B	Material constants	*E*_0_	Elastic modulus of model spring
*β*_T_	Coefficient of thermal expansion	*E*_1_	Elastic modulus of spring and fractional springpot
*T*_r_	Master curve reference temperature	*β*	Fractional order, 0 ≤ *β* ≤ 1
*T*	Experimental temperature (°C)	H(*τ*)	Relaxation time spectrum
*f*_0_	The free volume fraction of the material under *T*_r_.	*τ*_1_	Relaxation time of fractional springpot
*a_T_*	Time–temperature superposition horizontal shift factor *a*_T_ = *η*/*η*_0_, *a*_T_ = *τ*/*τ*_0_.	*τ*_2_	Relaxation time of fractional springpot
*η*_0_	Viscosity of the material under *T*_r_	*t*	Experimental time
log*a*_T_	Horizontal shift factor		

## References

[1] Liu M., Qiu Y., Shi Y., Qiu Y., Xu P., Chen X. (2024). Fabrication of flame retardant nylon 6 film composites with superior gas barrier property and puncture resistance. Compos. Commun..

[2] Artykbaeva E., Durmaz B.U., Aksoy Golshaei P.P., Aytac A. (2022). Investigation of the properties of PA6/PA610 blends and glass fiber reinforced PA6/PA610 composites. Polym. Compos..

[3] Li B., Liao G., Li Y., Yin H., Cui L., Cao K., Xie Z., Liu J., Liu Y. (2024). Investigation on the Correlation between Biaxial Stretching Process and Macroscopic Properties of BOPA6 Film. Polymers.

[4] Li-Jun S. (2020). Fractional derivative models for viscoelastic materials at finite deformations. Int. J. Solids Struct..

[5] Kanai T., Okuyama Y., Takashige M. (2018). Dynamics and structure development for biaxial stretching polyamide 6 films. Adv. Polym. Technol..

[6] Li B., Liao G., Liu J., Xie Z., Cui L., Yang Y., Liu Y. (2023). Investigation on The Tensile Rheological Behavior of PA6 Film Based on Fractional Order Model. Mater. Res. Express.

[7] Xu B., Blok R., Teuffel P. (2023). An investigation of the effect of relative humidity on viscoelastic properties of flax fiber reinforced polymer by fractional-order viscoelastic model. Compos. Commun..

[8] Butaud P., Ouisse M., Placet V., Renaud F., Travaillot T., Maynadier A., Chevallier G., Amiot F., Delobelle P., Foltête E. (2018). Identification of the viscoelastic properties of the tBA/PEGDMA polymer from multi-loading modes conducted over a wide frequency–temperature scale range. Polym. Test..

[9] Liu Y., Liao G., Liu Y., Li X., Liu X., Dong Y., Fu R., Fan S. (2021). Investigation on the creep behavior of PA6 film based on the fractional differential model. J. Elastomers Plast..

[10] Fahad H.M., Fernandez A. (2021). Operational calculus for Caputo fractional calculus with respect to functions and the associated fractional differential equations. Appl. Math. Comput..

[11] Dilmi M., Dilmi M., Benseridi H. (2021). Variational formulation and asymptotic analysis of viscoelastic problem with Riemann-Liouville fractional derivatives. Math. Method Appl. Sci..

[12] Zhu F., Xing G.H., Lyu G.J., Zhang L.T., Wang Y., Yang Y., Pelletier J.M., Qiao J.C. (2023). Physics-motivated fractional viscoelasticity model for dynamic relaxation in amorphous solids. Int. J. Plast..

[13] Koeller R.C. (1984). Applications of Fractional Calculus to the Theory of Viscoelasticity. Trans. ASME J. Appl. Mech..

[14] Han B., Yin D., Gao Y. (2023). The application of a novel variable-order fractional calculus on rheological model for viscoelastic materials. Mech. Adv. Mater. Struct..

[15] Zopf C., Hoque S.E., Kaliske M. (2015). Comparison of approaches to model viscoelasticity based on fractional time derivatives. Comp. Mater. Sci..

[16] Li X., Sha A., Jiao W., Song R., Cao Y., Li C., Liu Z. (2023). Fractional derivative Burgers models describing dynamic viscoelastic properties of asphalt binders. Constr. Build. Mater..

[17] Cai T., Feng Z., Jiang Y., Zhao D., Zhang X. (2019). Anisotropy Characteristics of Stress Relaxation in Coal: An Improved Fractional Derivative Constitutive Model. Rock Mech. Rock Eng..

[18] Alcoutlabi M., Martinez-Vega J.J. (1998). Application of fractional calculus to viscoelastic behaviour modelling and to the physical ageing phenomenon in glassy amorphous polymers. Polymer.

[19] Yin B., Hu X., Luo W., Song K. (2017). Application of fractional calculus methods to asymmetric dynamical response of CB-Filled rubber. Polym. Test..

[20] Li K.F., Yang C.Q., Zhao Y.B., Pan Y., Xu F. (2020). Study on the creep behavior of PVA-ECC based on fractional-differential rheological model. Constr. Build. Mater..

[21] Hooda N., Damani O. (2017). A System for Optimal Design of Pressure Constrained Branched Piped Water Networks. Procedia Eng..

[22] Williams M.L., Landel R.F., Ferry J.D. (1955). The Temperature Dependence of Relaxation Mechanisms in Amorphous Polymers and Other Glass-forming Liquids. J. Am. Chem. Soc..

[23] Laidler K.J. (1984). The development of the Arrhenius equation. J. Chem. Educ..

[24] Koomson C., Zeltmann S.E., Gupta N. (2018). Strain rate sensitivity of polycarbonate and vinyl ester from dynamic mechanical analysis experiments. Adv. Compos. Hybrid Mater..

[25] Hernández W.P., Castello D.A., Roitman N., Magluta C. (2017). Thermorheologically simple materials: A bayesian framework for model calibration and validation. J. Sound Vib..

[26] Lainé E., Bouvy C., Grandidier J.C., Vaes G. (2019). Methodology of Accelerated Characterization for long-term creep prediction of polymer structures to ensure their service life. Polym. Test..

[27] Matsuoka S. (1986). Free Volume, Entropy, and Relaxation Phenomena. J. Rheol..

[28] Kanakkanatt S.V. (1973). Application of the Cole-Cole Plot to Dynamic Data. J. Cell. Plast..

[29] Kaya S., Takolu N., Kolcu F. (2017). Synthesis and characterization of semi-conductive, thermally stable imine polymers containing methyl silane group. Polym. Bull..

[30] Yao D. (2018). A fractional dashpot for nonlinear viscoelastic fluids. J. Rheol..

[31] Thaijaroen W., Harrison A.J.L. (2010). Nonlinear dynamic modelling of rubber isolators using six parameters based on parabolic spring, springpot, and smooth-slip friction element. Polym. Test..

[32] Parmar R. (2015). A Class of Extended Mittag–Leffler Functions and Their Properties Related to Integral Transforms and Fractional Calculus. Mathematics.

